# An Exploratory Accounting Approach for Balancing Environmental Impacts of Point and Diffuse Sources in Nitrogen Trading

**DOI:** 10.1007/s00267-026-02626-7

**Published:** 2026-09-19

**Authors:** Jing Lu, Katherine R. O’Brien, Felix Egger, Tony Weber, Jon M. Olley, Matthew P. Adams, Michele A. Burford

**Affiliations:** 1 Australian Rivers Institute, Logan Campus, Griffith University, 68 University, Meadowbrook, QLD Australia; 2 School of Environment and Science, Griffith University, Brisbane, QLD Australia; 3 School of Chemical Engineering, Faculty of Engineering, Architecture and Information Technology, The University of Queensland, St Lucia, QLD Australia; 4 Alluvium, Fortitude Valley, QLD Australia; 5 Securing Antarctica’s Environmental Future, Queensland University of Technology, Brisbane, QLD Australia; 6 School of Mathematical Sciences, Queensland University of Technology, Brisbane, QLD Australia; 7 Centre for Data Science, Queensland University of Technology, Brisbane, QLD Australia

**Keywords:** Nutrient trading, Nitrogen offsetting, Point source pollution, Diffuse source pollution, Environmental equivalency, Watershed mitigation, Co-benefits

## Abstract

Nutrient trading between point and diffuse sources of nutrients offers an integrated watershed nutrient management approach to achieve nutrient reduction goals that benefit both the terrestrial and aquatic ecosystems. This market-based approach requires quantification of the environmental impacts of nutrients derived from different sources. This environmental impact depends on the forms, concentrations, loads, transport, and processing of nutrients within waterways. However, the impacts of source-specific nutrient characteristics in different point and diffuse nutrient sources have not been accounted for in nutrient trading schemes. Here, we explored an accounting method for nitrogen trading that accounts for different aquatic environmental impacts of point and diffuse sources. This environmental impact quantification is based on model fittings of a published dataset on algal photosynthetic responses to the key nitrogen form, i.e., total dissolved nitrogen rather than total nitrogen, from three sources: point source discharge from tertiary-treated sewage and aquaculture ponds, and diffuse watershed erosion runoff. We explored a method for decomposing trading ratios into environmental equivalency, delivery ratio, and uncertainty component for different sources, and considering the co-benefits of watershed mitigation. Our results demonstrate that prioritizing diffuse nitrogen sources with a greater aquatic environmental impact and co-benefits can significantly reduce the watershed mitigation requirements for offsetting the environmental impacts of point-source nitrogen loads. This study presents an exploratory accounting approach for nitrogen trading that incorporates the differential environmental impacts of point and diffuse nutrient sources on aquatic ecosystems. Alternative approaches to equating environmental impacts across sources with different nutrient characteristics also warrant further investigation.

## Introduction

In many developed countries, nitrogen and phosphorus pollution from point sources has decreased markedly over recent decades due to technological advances in wastewater treatment and increased regulation (Frei et al., 2021). Consequently, nutrient loads from diffuse sources, such as soil erosion, are becoming more dominant in many watersheds (Howarth et al., 2002; Nie et al., 2018). Due to diminishing returns, further sewage treatment plant (STP) upgrades may not be the most cost-effective way to reduce nutrient impacts on receiving waters. Nutrient trading schemes offer a cost-effective way to tackle nutrient pollution and drive investment in diffuse nutrient load reduction (Selman et al., 2009). These schemes often involve point-source dischargers offsetting their emissions by reducing diffuse nutrient loads elsewhere. For example, diffuse nutrient loads can be reduced via watershed mitigation actions, such as soil erosion control and improved fertilizer management, as an alternative to expensive treatment plant upgrades (Shortle, 2012). A range of watershed mitigation actions can be classified as nature-based solutions. Nature-based solutions are “actions to protect, conserve, restore, sustainably use and manage natural or modified terrestrial, freshwater, coastal and marine ecosystems which address social, economic and environmental challenges effectively and adaptively, while simultaneously providing human well-being, ecosystem services, resilience and biodiversity benefits” (UNEA, 2022). Common nature-based solutions in watershed management include riverbank stabilization, revegetation, riparian restoration, and wetland restoration. These mitigations can reduce nutrient and sediment loads while also providing broader environmental benefits, such as enhanced biodiversity and carbon sequestration (Olson et al., 2007; Fortier et al., 2015). Therefore, trading between point and diffuse sources of nutrients offers an integrated watershed management approach to achieve nutrient reduction goals that benefit both the terrestrial and aquatic ecosystems.

To ensure minimal impacts on receiving waters, nutrient trading for point sources must comply with effluent limits and can only be considered when other on-site options for nutrient treatment are insufficient or unfeasible. The adoption of nutrient trading has been hindered by social, economic, institutional, and policy challenges, particularly in developing the trading rules and institutions that can produce effective economic and ecological gains (King and Kuch, 2003; Fisher-Vanden and Olmstead, 2013; Shortle et al., 2021). There are also biophysical-chemical knowledge gaps that hinder the implementation of nutrient trading schemes (Lu et al., 2023). For example, there is a range of uncertainties related to nutrient trading between point and diffuse sources (Horan and Shortle, 2005; Arabi et al., 2007; Hoag et al., 2017), including spatial and temporal variability in nutrient export from diffuse sources, the effectiveness of nutrient reduction from mitigation of diffuse sources, and the estimation of transport and processing of nutrients from diffuse sources along the river from upstream to downstream. To account for those uncertainties in nutrient trading programs, a trading ratio greater than one (>1) is commonly applied. A trading ratio defines the amount of nutrient reduction required from a nitrogen credit seller to offset one unit of nutrient discharge from a credit buyer. In this study, credit refers to a quantified nitrogen reduction unit generated by a mitigation action. For example, a 2:1 trading ratio requires two units of nutrient reduction from the seller for every one unit of nutrient released by the buyer. In previous studies, trading ratios typically range from 1.5:1 to 4:1 (Morgan and Wolverton, 2005; Hoag et al., 2017), requiring diffuse sources to reduce 1.5 to 4 units of nutrients for every unit released by a point source. While a higher trading ratio can minimize negative impacts on aquatic ecosystems, it may also disincentivize participation in nutrient trading and investment in watershed mitigation. Keller et al., (2014) presented a systematic approach to determining attenuation coefficients and their associated uncertainty for total nitrogen (TN) and total phosphorus (TP) transport and processing in rivers based on travel distance and river orders, providing a scientific basis for part of the trading ratio quantification. However, there is limited scientific evidence to support the quantification of the environmental equivalency between point and diffuse sources on aquatic systems (Puzyreva et al., 2019; Lu et al., 2023).

Nutrient trading relies on the neutrality of impacts on receiving waters, such that buyers must offset their nutrient discharge impacts by funding equal or greater reductions from the sellers (U.S. EPA 2003). Most nutrient trading schemes assume that total nutrient loads, such as TN, have equal environmental impacts regardless of source (Stephenson and Shabman, 2017; Boleman and Jacobson, 2021). However, the concentration and form of nutrients delivered from different sources can affect the scale of environmental impact. Lu et al., (2024a, b, 2026) used algal photosynthetic yield response as a proxy for aquatic environmental impacts of point and diffuse nitrogen sources to conclude that the best predictor of this environmental impact was the concentration of total dissolved nitrogen (TDN) rather than TN.

**Fig. 1:**
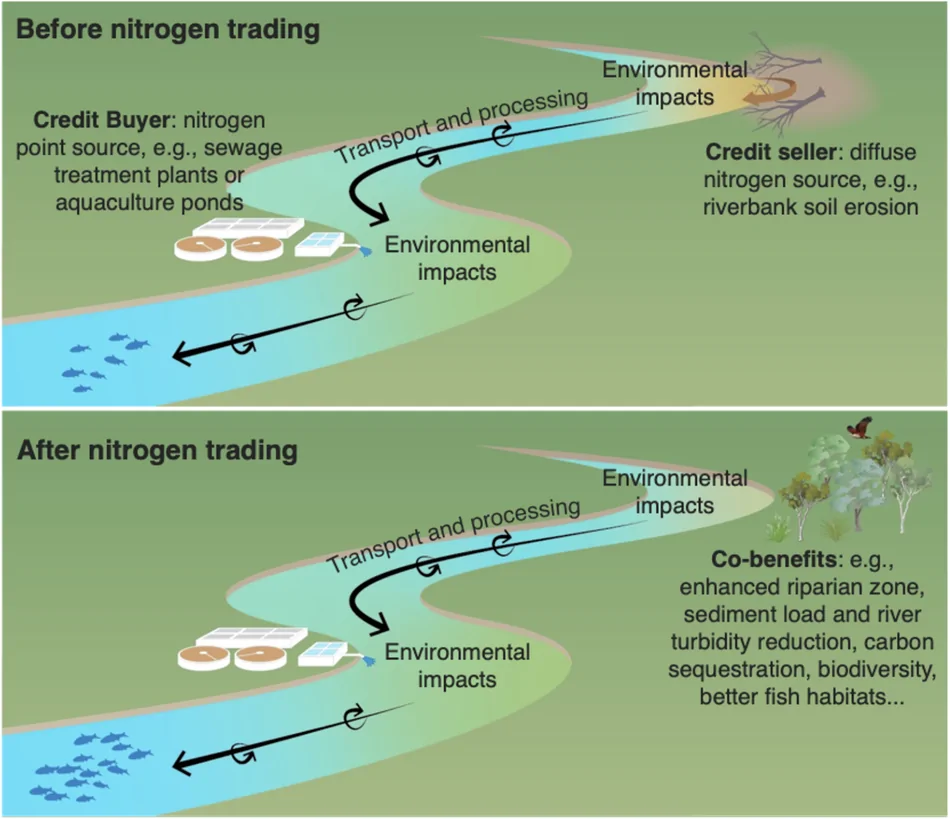
Conceptual illustration of the nitrogen trading example, which equates the environmental impact of different nitrogen sources delivered at different locations. The credit buyer, such as a sewage treatment plant or an aquaculture pond, needs to reduce elsewhere environmental impacts of nitrogen discharge. The credit seller reduces nitrogen input during flow events via riverbank mitigation

The present work builds on the findings and dataset from Lu et al. (2024a, b, 2026) to explore a potential accounting method, specifically for nitrogen trading, between point and diffuse sources within the same watershed. This exploratory approach aims to equate the environmental impacts of point and diffuse sources on receiving waters. As an illustrative case, we consider the common example of a downstream point source, such as an STP or aquaculture pond farm, acting as a credit buyer, offsetting its environmental impacts via a nitrogen load reduction in an upstream diffuse source. In this example, the credit seller implements measures such as riverbank soil erosion mitigation (Fig. 1). This exploratory accounting method calculates the seller’s required nitrogen reduction by accounting for differences in environmental impacts between point and diffuse sources. We acknowledge the policy and institutional challenges in implementing market-driven nutrient trading schemes discussed in previous studies (Stephenson and Shabman, 2017; Shortle et al., 2021). In this context, the present work provides an exploratory accounting approach to illustrate how such a method may be structured to improve confidence among regulators and investors that trading can minimize the risk of unintended environmental impacts, based on a solid scientific foundation.

Therefore, the main research question addressed in this study is: How can nitrogen trading account for differences in aquatic environmental impacts between point and diffuse nitrogen sources? Specifically, we aimed to develop an exploratory accounting method for determining source-specific environmental equivalency in nitrogen trading and to demonstrate its application using an illustrative point-diffuse nitrogen trading case study. The case study compares different accounting scenarios, including the proposed exploratory method and a baseline approach based on conventional nitrogen load accounting.

## Exploratory Accounting Method for Nitrogen Trading

### Key Aspects of the Accounting Method

To ensure that environmental impacts of nitrogen discharge from buyers are sufficiently offset by engaging sellers, the forms and concentrations of nitrogen from each source need to be considered. Here, we use the term Equivalency Ratio (*ER*) to capture these differences. Since nitrogen markets typically work by trading nitrogen increases at one location with nitrogen reductions at a different location (Fig. 1), the transport and processing of nitrogen within waterways must also be considered. This transport and processing of nitrogen is captured using a Delivery Ratio (*DR*). To equate the environmental impacts across nutrient sources, co-benefits and uncertainty should also be considered for their contribution to nitrogen trading. In this exploratory approach, the trading ratio is separated into the environmental equivalency ratio (*ER*), delivery ratio (*DR*), uncertainty (*UF*) and co-benefits components (*BF*). Combining all the above considerations, we propose that the nitrogen load reduction required by a credit seller upstream, i.e., *Ns* (t), from a point source credit buyer downstream is calculated as

1 $${N}_{s}={N}_{b}\,\times {ER}\times {DR}\times {BF}\times {UF}$$

Where *Nb* is the nitrogen load (t) from the credit buyer for trading, *BF* is the Co-benefits Factor (unitless), and *UF* is the Uncertainty Factor (unitless).

We considered *ER*, *DR*, *BF*, and *UF* because together they represent four major biophysical and accounting considerations required to compare nitrogen impacts across sources. *ER* accounts for differences in aquatic environmental impacts per unit nitrogen from different sources. *DR* accounts for changes in nitrogen load or effect during transport between seller and buyer locations. *BF* recognizes additional environmental benefits associated with mitigation actions. *UF* provides a safety margin for uncertainty in load estimation, mitigation performance, and ecological response. Other factors, such as temporal credit generation, market governance, compliance monitoring, and institutional trading rules, are also important for implementation, but were not included as explicit parameters in Eq. (1) because the focus of this study is the biophysical accounting basis for comparing nitrogen impacts. Specifically, the present work focuses on providing a scientifically robust derivation of *ER*, based on data of Lu et al. (2024a, b, 2026), between point and diffuse sources in the trading example shown in Fig. 1. It also considers the definition of *DR*, *BF*, and *UF* with default values due to a lack of knowledge and limited site-specific evidence, requiring further research. The application of the accounting method to our example (Section below) describes each of the five factors in Eq. (1) in detail.

### Study Context and Nutrient Sources

Details of the point and diffuse nitrogen sources used to develop this exploratory accounting method are provided in Lu et al. (2024a, b, 2026). Briefly, the point-source examples were effluents from STPs and aquaculture farms (both fish and prawn farms). These samples were collected from the north Queensland, southeast Queensland, and Sydney regions of eastern Australia, where STPs and aquaculture facilities represent important regulated point sources of nitrogen to receiving waters. The diffuse-source example represents nitrogen derived from riverbank soil erosion, which is a significant contributor to diffuse nutrient loads in many Australian watersheds (Olley et al., 1996, 2013; Brooks et al., 2014) and an overlooked diffuse nutrient source in U.S. watersheds (Margenot et al., 2023). In Lu et al. (2024a, b, 2026), diffuse-source nitrogen was represented using laboratory-simulated erosion runoff, referred to as soil slurries, following the method of Garzon-Garcia et al. (2018). These soil slurries were prepared from soils collected across five eastern Australian watersheds: the Lockyer, Brisbane, Logan, and Bowen River watersheds in Queensland, and the Hawkesbury–Nepean River watersheds in New South Wales. Soil sampling sites included gullies, riparian buffers, grazing paddocks, and forested areas, thereby capturing a range of land-use conditions relevant to erosion-derived nutrient inputs.

This example context reflects a common point–diffuse nutrient trading scenario in Australia, where downstream point-source dischargers may offset nitrogen impacts through upstream soil erosion mitigation. The dataset in Lu et al. (2024a, b, 2026), therefore, provides a practical basis for exploring how source-specific nitrogen characteristics can be incorporated into a nitrogen trading accounting method.

## Application of the Accounting Method

We illustrate the five factors in Eq. (1) using the example where a downstream point source, such as an STP or aquaculture pond, offsets its environmental impacts via an upstream diffuse source of riverbank soil erosion mitigation (Fig. 1).

### The Nitrogen Load From the Credit Buyer for Trading (Nb)

*N*_*b*_ (t) is calculated as the point source nitrogen concentration multiplied by discharge and summed over the trading period (time *i* = 1 to n)

2 $${N}_{b}={\sum}_{i=1}^{n}{\left({C}_{i}{D}_{i}\right)}\times {10}^{-9}$$

Where *C*_*i*_ is the concentration of nitrogen (mg L^-1^) in the point source discharge at time *i*, and *D*_*i*_ is the buyer’s discharge (L) at time *i*. The factor ${10}^{-9}$ is the unit conversion of mg to t.

### Equivalency Ratio (ER)

The form of nitrogen released often differs between different sources, and this is accounted for in Eq. (1) with an Equivalency Ratio (*ER*). Currently, there is no standardized method for comparing the aquatic environmental equivalency of different nitrogen sources. The simplest approach assumes all nitrogen from any given source, irrespective of its initial form, is ultimately bioavailable over time. In this case, the nitrogen trade would be based on TN and *ER* = 1. If *ER* = 1, the nutrients released by the seller and buyer generate equivalent environmental impacts. If *ER* < 1, nitrogen release by the buyer has less impact on receiving waters, so less nitrogen load from the seller is required to offset the buyer’s impact. Conversely, if *ER* > 1, the buyer’s nitrogen form has more impact; therefore, it requires a greater load from the seller to offset the impact of the buyer. We next present a scientifically defensible procedure for deriving an expression of *ER*, using fitted empirical models on a published dataset from Lu et al. (2024a, b, 2026).

**Fig. 2:**
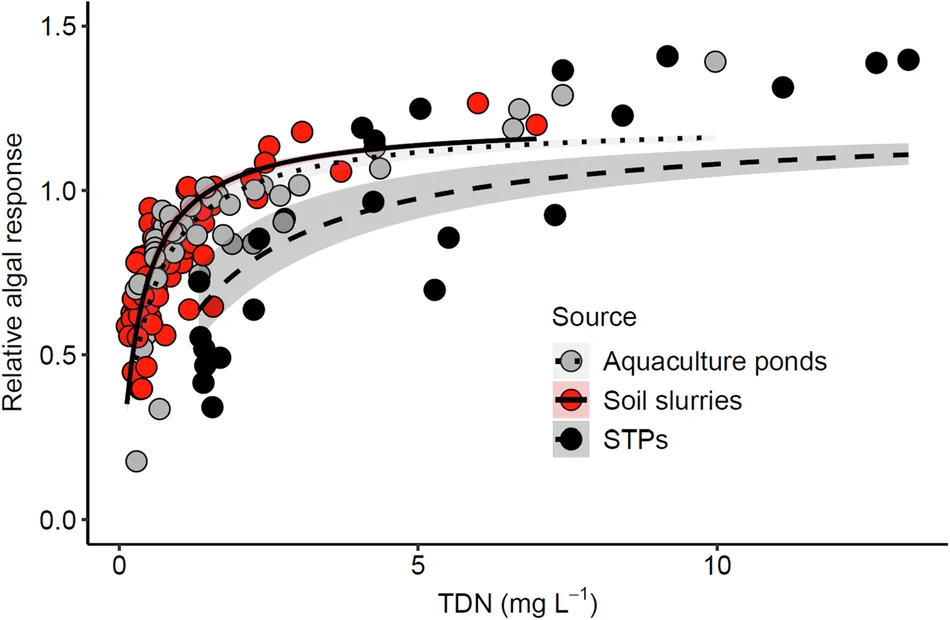
Relationships between the relative algal response (dimensionless) and the total dissolved nitrogen concentration TDN (mg L^-1^), parameterized using Eq. (3), from STPs (black dots with dashed black curve), aquaculture ponds (grey dots with dotted black curve), and soil slurries (red dots with solid black curve). Lines represent fitted curves, and shaded ribbons indicate the corresponding 95% confidence intervals. The data are from Lu et al. (2024a, b), and the figure is adapted from Figure 7 in Lu et al (2026). The dataset comprised 24 STP effluent samples, 45 aquaculture pond samples, and 90 soil slurry samples, including some repeated samples collected from the same sites or point sources in Queensland and New South Wales, Australia

Lu et al. (2024a, b, 2026) recently demonstrated that the concentration and form of nitrogen from different sources can have different environmental impacts, with total dissolved nitrogen (TDN) being the best predictor for algal photosynthetic yield response. Their studies used a standardized three-day algal bioassay to measure the photosynthetic response of a nitrogen-starved freshwater algal species to nutrients derived from Australian nitrogen point sources (STPs and aquaculture ponds) and eroded soil runoff over a range of concentrations (soil slurries). Soil slurries were processed from soils to simulate soil erosion and sediment transport from land to water - see Lu et al. (2023b, 2024a) and Garzon-Garcia et al. (Garzon-Garcia et al., 2018) for full technical details. The data collected by Lu et al. (2024a, b) are presented in Fig. 2 (revised from Figure 7 in Lu et al. (2026)), showing that TDN from STPs elicits a lower algal photosynthetic response for a given concentration of TDN than that from the soils and aquaculture ponds.

In the present work, Michaelis-Menten models (curve lines in Fig. 2), which are commonly used to describe the algal response to available nutrients (Hoppe et al. 1988), are fitted to each nutrient source individually as a critical step towards deriving an expression for *ER* required in Eq. (1). Denoting *P*_*i*_ as the relative algal photosynthetic yield response (dimensionless) and *N*_*i*_ as the nitrogen concentration (mg L^-1^) for some nitrogen source *i* as a nitrogen credit seller or buyer: the different sources illustrated in Fig. 2. The Michaelis–Menten model was selected because algal response to nutrient concentration commonly follows a Monod-type equation, where response increases strongly at low nutrient concentrations but approaches a maximum at higher concentrations. Michaelis-Menten kinetics was originally used as a model that describes the rate of enzymatic reactions, where the reaction rate is saturable and not solely proportional to substrate concentration, with an equation that incorporates maximum velocity and a Michaelis constant (Cornish-Bowden, 2013). In more recent years, it has been applied to algal physiology and used in models predicting algal growth or photosynthetic responses to nutrient concentrations (Wang et al. 2022; Lu et al., 2024b, 2026). The model provides a biologically interpretable half-saturation constant, *K*, which indicates the nitrogen concentration required to achieve half of the maximum response. This feature makes the model suitable for comparing the relative environmental effects of nitrogen from different sources and for deriving ER. The Michaelis-Menten model for relative algal photosynthetic yield vs. nitrogen concentration is

3 $${P}_{i}={P}_{{maxi}}\frac{{Ni}}{{Ni}\,{+K}_{i}}$$

Where *P*_*maxi*_ is the maximum achievable algal response (dimensionless) and *K*_*i*_ is the half-saturation constant that represents the nitrogen concentration (mg L^-1^) that is expected to yield half of *P*_*maxi*_, i.e., *P*_*i*_ = ½ *P*_*maxi*_. Subscripts *i* in *P*_*maxi*_ and *K*_*i*_ refer to the nitrogen source, either a credit seller or buyer.

In fitting the Michaelis-Menten model for algal response to the three nitrogen sources, we assume that *P*_*maxi*_ (the maximum achievable algal response) is the same for all nitrogen sources, as this assumption appeared to be very plausible for the data available (see Fig. 2). This means that all nitrogen sources will reach the same *P*_*max*_ for the algal growth when the TDN concentration is sufficiently high, so that

4 $${P}_{{maxi}}={P}_{\max }$$

For the *ER* derivation, the buyer and seller need to have the same environmental impacts, i.e., the same algal photosynthetic yield response in the present work, so that

5 $${P}_{{seller}}={P}_{{buyer}}$$

The definition of *ER* in Eq. (1) shows that

6 $${ER}=\frac{{N}_{{seller}}}{{N}_{{buyer}}}$$

Where *N*_*seller*_ or *N*_*buyer*_ is the nitrogen concentration from the seller or the buyer.

Therefore, Eqs. (3), (4), and (5) imply

7 $${ER}=\frac{{N}_{{seller}}}{{N}_{{buyer}}}=\frac{{K}_{{seller}}}{{K}_{{buyer}}}$$

Where *K*_*seller*_ or *K*_*buyer*_ is the half-saturation constant for the seller or the buyer in Eq. (3).

**Fig. 3:**
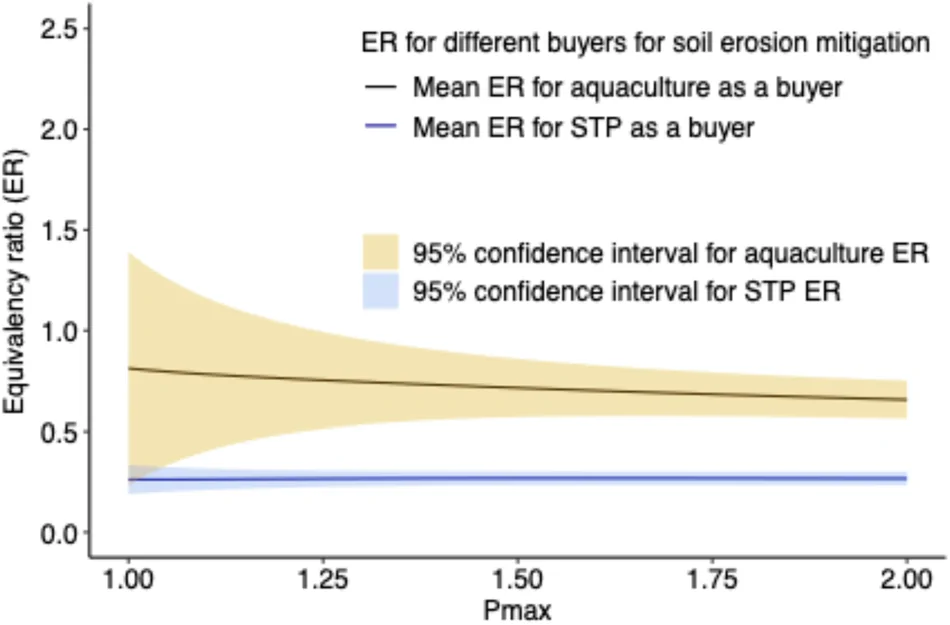
The changes of Equivalency ratio (ER) in Eq. (7) for nitrogen trading between STP and soil erosion mitigation or between aquaculture ponds and soil erosion mitigation, when P_max_ (the maximum achievable algal response) in Eq. (3) changes from 1 to 2

We test a range of *P*_*max*_ values ranging from 1 to 2 to investigate the *P*_*max*_ effect on *ER* uncertainties. The results indicate that changes in *P*_*max*_ from 1 to 2 have little impact on the *ER* for the buyer when trading with soil erosion control. For example, if the buyer is discharging from aquaculture ponds, the mean *ER* varies between 0.68 and 0.86; if the discharge is from STPs, the mean *ER* varies between 0.26 and 0.27 (Fig. 3). This indicates that the change of *P*_*max*_ does not significantly impact *ER* calculations. However, to achieve a minimum uncertainty in the model fitting for all nitrogen sources, *P*_*max*_ was set to 1.21 for all nitrogen sources in Fig. 2 and Table 1. The optimal model fitting was identified by a lower root-mean-square error (RMSE) and a higher R^2^, indicating a lower uncertainty in the model performance. RMSE represents the average magnitude of prediction error for the fitted model in the same units as the response variable, and R^2^ quantifies the proportion of variation in observed algal response that can be explained by the fitted model. The standard error was also reported in Table 1 to provide an estimate of the uncertainty associated with the fitted parameter value, *K*_*i*_, which represents the source-specific half-saturation constant. The validity of this model fitting is likely stronger when TDN concentration was below 5 mg L^-1^ due to the limited data points at higher concentrations (Fig. 2).

**Table 1:** The Michaelis-Menten model fitting statistics (R^2^ and RMSE) and the half-saturation constant (Ki), together with the associated 95% confidence intervals for Eq. (3), when P_max_ = 1.21 for the three nitrogen sources

Nitrogen sources	Typical trading role		No. of samples	Half-saturation constant *Ki* (mg L^-1^)	R^2^	RMSE
Sewage treatment plants (STPs)	Buyer	24		1.20 ± 0.14	0.58	0.22
Aquaculture ponds	Buyer	45		0.42 ± 0.14	0.70	0.13
Soil slurries (a proxy for watershed erosion)	Seller	90		0.32 ± 0.03	0.50	0.13

Nutrient source samples were taken from twenty different STPs, fifteen aquaculture ponds, and twenty-three different soil sites, with repeated samples for each source from sites in southeast Queensland and New South Wales areas. This table is adapted from Table 2 in Lu et al (2026)

### Delivery Ratio (DR)

In our example of trading between an upstream diffuse-source credit seller and the downstream point-source credit buyer (Fig. 1), the transport and within-waterway processing of nitrogen between these two locations need to be considered (Saby et al., 2021). In an Australian example, where nitrogen sources were collected in Lu et al. (2024a, b, 2026), analysis of long-term monitoring data for the Brisbane River estuary revealed that TN loads from large flood events predominantly passed through without detectable removal (Newham et al., 2024). In our example of trading point sources with soil erosion-derived nitrogen, we assume negligible TDN processing between seller and buyer sites. This assumption is likely to be valid because our example assumes erosion-derived nitrogen export during large rainfall or flood events, when water residence time is short within a river or creek system, and in-stream TDN processing between the seller and buyer is likely to be limited. This differs from attenuation-coefficient approaches, such as Keller et al. (2014), which estimate attenuation of total nutrients as a function of river travel distance and stream order, rather than specifically addressing TDN as in our example. These approaches from Keller et al. (2014) are particularly useful when sufficient information is available to parameterize spatial total nutrient processing. The advantage of using DR = 1 is transparency and simplicity for an exploratory accounting example. However, it may overestimate nitrogen reductions at the seller’s site where substantial in-stream nitrogen uptake, denitrification, or transport and processing occur. In addition, nitrogen loading from major floods can also affect coastal receiving waters in the longer term due to sediment resuspension and remineralization processes (Grinham et al., 2024). This may lead to TDN release under different flow and receiving water conditions, resulting in Delivery Ratio (*DR*) < 1. In the absence of other information, we here set the Delivery Ratio to its default value of *DR* = 1, but we acknowledge that if robust modelling or field data demonstrate nitrogen processing, the default value of *DR* should be replaced with the best available estimate, such as those derived from modelling studies of nutrient attenuations for total nutrients in U.S. rivers (Keller et al., 2014).

### Co-Benefit Factor (BF)

Actions of watershed mitigation by credit sellers to reduce nutrient loads can also provide co-benefits for aquatic and terrestrial environments, including sediment pollution reduction, enhancement in carbon sequestration, biodiversity, flood mitigation, habitat connectivity, and groundwater recharge (Fortier et al., 2015; Dixon et al., 2016). Attributing value to co-benefits in nutrient accounting provides a fairer assessment of sellers’ actions, incentivizes investments in nature-based solutions to address environmental challenges, and supports broader-scale environmental benefits (White et al., 2020; Limb et al., 2024). There is increasing interest in stacking co-benefits within environmental markets, but practical examples remain limited (Shortle et al., 2021). Given the evolving nature of co-benefit assessment in natural capital markets (White et al., 2020; Brandon et al., 2021), our nitrogen accounting methodology did not quantitatively assess these co-benefits for nitrogen trading. However, we explicitly include a dimensionless Co-benefit Factor (*BF*, default value = 1) in Eq. (1) to encourage recognition and quantification of these valuable co-benefits. If any co-benefits are recognized, the Co-benefit Factor *BF* will be less than 1, indicating that less nitrogen load is required from the watershed mitigation to offset nitrogen release from the buyer.

### Uncertainty Factor (UF)

Nitrogen markets rely on investor confidence in protecting environmental values, which depends on accurately equating nitrogen impacts from credit buyers and sellers. However, the inherently complex, dynamic, and variable nature of aquatic ecosystems makes it difficult to assign causality (O’Brien et al. 2018). Thus, the nutrient market needs a margin of error in determining trading credits to account for uncertainty in estimating nutrient loads from diffuse sources and watershed mitigation effectiveness. This margin of error can also cover the uncertainties related to the longer transport time lags that may occur in diffuse sources due to their movement through soils, groundwater, etc., compared to point sources (Cook and Shortle, 2022). Our nitrogen accounting methodology includes an uncertainty factor. This practical approach adopts the assumption that the margin of error is directly proportional to the amount of nutrients being traded, rather than following more complex patterns as discussed in other studies (Horan, 2001; Horan and Shortle, 2017). Multiple uncertainties may vary nonlinearly with hydrological conditions, lag times between mitigation implementation and water quality responses, spatial variability in diffuse-source loads, and the probability of mitigation failure. In our illustrative method, *UF* is represented as a simple multiplier because insufficient evidence is available to define a dynamic uncertainty function. We recommend a default value ranging from 1.5 to 4, following the previously mentioned trading ratios assumed in U.S. studies. This *UF* range is not intended to represent differences in source-specific nitrogen environmental impact, as those differences are addressed separately through ER. Instead, this range is used as an illustrative safety margin for uncertainty associated with diffuse-source load estimates, mitigation effectiveness, monitoring limitations, and temporal performance, etc. In this accounting method, we also assume that mitigation works are maintained and remain effective over the trading period. Therefore, this value should be reduced with evidence from individual projects, such as sophisticated modelling or field sampling, as well as continuous monitoring and maintenance needed for mitigation projects. For example, at the proposal stage, investment in nutrient export estimates from agricultural farmland through the development and validation of predictive models, as well as the quantification of nutrient losses associated with soil erosion from riverbanks during storm events, can substantially reduce the uncertainty factor. There is a clear financial incentive to reduce uncertainty as it allows more feasible nutrient trading schemes, which subsequently will incentivize participation. In contrast, where source-specific nutrient characteristics, mitigation effectiveness, or nutrient transport and processing processes are poorly constrained, a higher UF, credit suspension or reserve mechanism, or additional site-specific assessment may be required (U.S.EPA 2007).

### Scenarios of Soil Erosion Mitigation Calculations for a Case Study

To illustrate the impacts of using TDN, rather than TN, and an *ER* of 0.3, rather than 1 (Fig. 3), we examine three theoretical scenarios for offsets (Table 2). In these scenarios, we calculate the scale of riverbank erosion mitigation works needed, based on Eq. (1), for a case study to offset 10 t of TN per year from the Oxley STP, which discharges into the mid-Brisbane River estuary. The three scenarios are:

1. TN load and *ER* = 1 scenario (baseline scenario): nitrogen loads for trading are calculated using TN and *ER* = 1, a common approach used in many nitrogen trading projects.
2. TDN load and *ER* = 1 scenario: nitrogen loads for trading are calculated using TDN, based on our findings that TDN is a better parameter than TN to compare environmental impacts. This scenario assumes TDN from all sources has the same environmental impact, i.e., *ER* = 1.
3. TDN load and *ER* = 0.3 scenario: The trading nitrogen load is also calculated using TDN, but *ER* = 0.3 based on our findings in Fig. 3.

**Table 2:** A comparison of the calculated soil stabilization (to reduce soil erosion) needed per year for offsetting 10 t per year of TN from Oxley STP

Scenarios		Scenario 1: TN load and ER = 1	Scenario 2: TDN load and ER = 1		Scenario 3: TDN load and ER = 0.3	
Eq. (1)*:* (default values: *DR* = 1, *BF* = 1, and *UF* = 1.5)	Loads for trade	TN	TDN		TDN	
	*Nb*: STP TN to trade (per year)	10 t	10 t		10 t	
	STP: TDN/TN (Fig. 4)	−	94%		94%	
	Equivalency ratio (ER)	1	1		0.3	
	Soil TDN needed (per year)	−	14.1 t		4.23 t	
	Soil: TDN/TN (Fig. 4)	−	2%	20%	2%	20%
	Soil TN needed (per year)	15 t	705 t	70.5 t	211 t	21 t
	Soil TN by weight	0.1%	0.1%	0.1%	0.1%	0.1%
	Soil density (t m^-3^)	~1.5	~1.5	~1.5	~1.5	~1.5
	Soil stabilization needed (per year)	15,000 t or ~10,000 m^3^	705,000 t or ~470,000 m^3^	70,500 t or ~47,000 m^3^	211,500 t or 141,000 m^3^	21,150 t or ~14,100 m^3^

This is based on Eq. (1) for three scenarios, using TN or TDN loads, two equivalency ratios (ER), and two proportions of TDN from TN in soils.

**Fig. 4:**
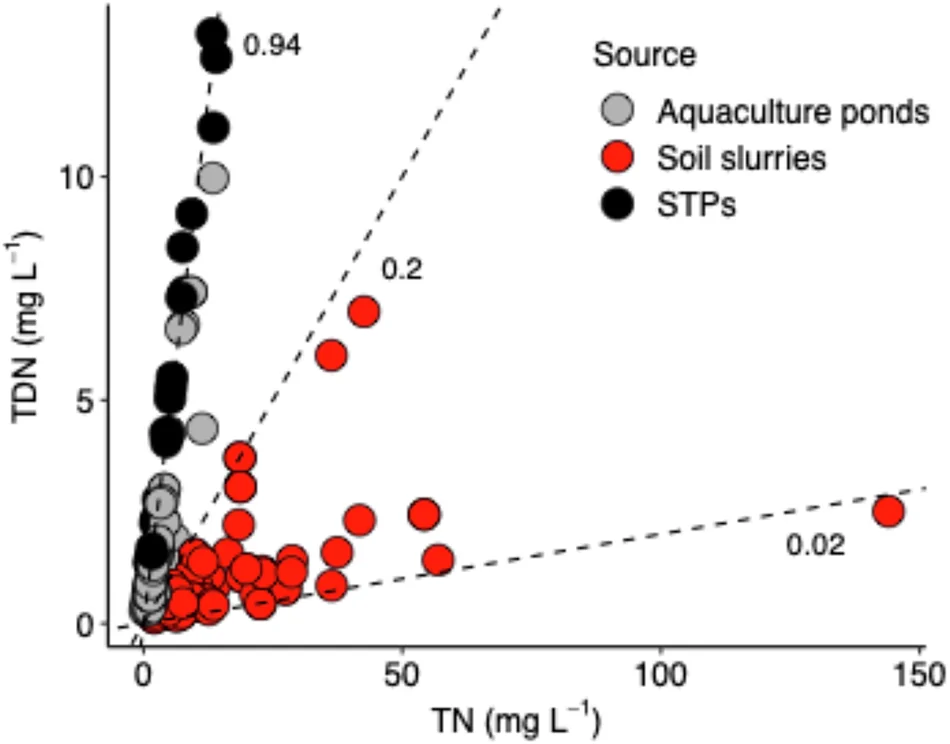
TDN vs TN concentrations (mg L^-1^) for the samples from aquaculture ponds, soil slurries and STPs used in Fig. 2. The data are from Lu et al. (2024a, b)

In the TDN scenarios (scenarios 2 and 3), the proportion of TDN from TN, i.e., TDN/TN, needs to be considered. Figure 4 shows that the proportion of TN present as TDN differs substantially among sources (Fig. 2). Samples from STPs and aquaculture ponds were dominated by dissolved nitrogen, with approximately 94% of TN present as TDN. In contrast, soil-derived samples had much lower and more variable TDN/TN proportions, ranging from approximately 2% to 20% (Fig. 4). This difference is important for nitrogen trading because TDN was identified as the nitrogen metric most closely associated with algal photosynthetic response. Therefore, mitigation sites with higher TDN/TN proportions may provide greater aquatic environmental benefit per unit of soil TN stabilized. Figure 4 provides the empirical basis for the TDN/TN assumptions used in the scenario calculations in Table 2.

For all three scenarios, a riverbank soil TN concentration of 0.1% and a soil density of 1.5 t m^–3^ are used for soil erosion mitigation calculations, and default values of 1, 1, and 1.5 are used for *DR*, *BF*, and *UF*, respectively, for each scenario. Because *DR*, *BF*, and *UF* enter Eq. (1) as a multiplier, higher *DR*, *BF*, and *UF* values would proportionally increase the required mitigation quantity across all scenarios, rather than affecting any single scenario. We used *UF* = 1.5 as the lower-bound illustrative value in this case study, consistent with values adopted in previous nutrient trading case studies in Queensland, Australia, to represent a case where uncertainty is partly reduced through site-specific information, riverbank erosion modelling, and mitigation design and maintenance efforts. This choice allows the accounting implications of TDN and ER to be demonstrated without compounding the example with a highly conservative uncertainty multiplier. However, we acknowledge that higher *UF* values may be appropriate where diffuse-source loads and mitigation effectiveness are poorly constrained.

To offset 10 t yr^-1^TN from Oxley STP, the TN load and *ER* = 1 scenario, i.e., the baseline scenario, requires 15 t yr^–1^ TN from riverbank mitigation (Table 2); the TDN load and *ER* = 1 scenario requires 70.5 to 705 t yr^–1^ TN from riverbank mitigation when soil TDN/TN varies between 20% and 2%, which is 4.7 to 47 times the soil TN needed than the baseline scenario. In contrast, the TDN load and *ER* = 0.3 scenario requires 21 to 211 t yr^–1^ TN from riverbank mitigation when soil TDN/TN varies between 20% and 2%, which is 1.4 to 14 times the soil TN needed in the baseline scenario.

Overall, scenarios of using TDN loads require more work to mitigate riverbank erosion than the baseline scenario of using TN as the nutrient metric for trading in most nitrogen trading examples (U.S. EPA, 2003; Selman et al., 2009; Shortle, 2012). This is because only a relatively lower proportion of TN exported from eroded soil is present in dissolved form, compared to STPs. However, the results also show that the scale of mitigation work required is highly sensitive to two key factors: ER and the TDN/TN proportion. A lower ER and a higher TDN/TN proportion in the mitigated soil can significantly reduce the mitigation work needed to offset the same TN loads from the STP. These findings suggest that nitrogen trading schemes should not treat all erosion mitigation sites as equivalent. Instead, mitigation should be prioritized towards sites that generate greater environmental benefits, consistent with watershed-scale prioritization approaches that aim to achieve pollutant reduction targets in the most cost-effective manner (Hermoso et al., 2015; U.S.EPA, 2018; Rohith et al., 2024). Prioritizing soil erosion sites with greater aquatic environmental impacts and a higher TDN/TN proportion can significantly reduce the investment in soil erosion mitigation requirements for nitrogen trading.

## Scope of Application and Further Research

Nutrient trading involving diffuse sources can result in broader-scale environmental benefits, but robust and scientifically sound accounting methods are needed to ensure environmental equivalency between point and diffuse nutrient sources. Our study showed that equivalency may be improved through the use of the nutrient metric, TDN, rather than TN, drawing on algal bioassay findings reported in Lu et al. (2024a, b, 2026). This accounting method with the derived ER is most applicable where trading is implemented between a point source and a diffuse source with different TN bioavailability, and where TDN is a relevant indicator of aquatic environmental response from different nutrient sources. Under conditions where this accounting method is not applicable, *ER* may be estimated using local algal or other relevant bioassays to test ecological responses to nutrient forms and concentrations, or ecological response models, provided they are calibrated for the local environment. *DR* may be estimated using attenuation coefficients or coupled hydrological and biogeochemical models; *BF* may be estimated using natural-capital or ecosystem-service assessment methods, and UF may be adjusted using project-specific uncertainty analysis. The *ER* derived in this study represents the source-specific biological effect per unit TDN under standardized bioassay conditions. It does not directly account for temporal differences in nitrogen loading, such as continuous STP discharge versus event-driven erosion-derived inputs, and the lag time for nutrient reduction from mitigation actions, which is also a key knowledge gap identified in previous studies (Lu et al., 2023). In practical trading schemes, temporal differences should be addressed through additional crediting rules, such as longer trading periods (seasonal, annual, or even decadal), event-based nutrient transport and processing assumptions, or dynamic trading ratios (Shortle et al., 2016; Cook and Shortle, 2022). Where credits are purchased before erosion mitigation reaches full effectiveness, the credit quantity could be discounted, delayed, or assigned a higher uncertainty factor until monitoring demonstrates that the mitigation action is functioning as expected (U.S.EPA, 2007).

It is important to note that the reported study used a single indicator of environmental impacts, i.e., algal growth response, to quantify the environmental equivalency of nutrients from different sources. While this assay provides an important biological response measure of nutrient impacts on aquatic ecosystems, it does not capture other key measures of impact, such as a reduction in biodiversity and hypoxia. For example, research by (Lu et al. 2025) showed that water column oxygen demand in estuaries can be differentially impacted by different nutrient sources, with organic carbon forms and concentrations in these sources being of particular importance. Future research could, therefore, aim to incorporate a broader suite of ecological indicators to develop a more holistic assessment of environmental equivalency. However, expanding the processes measured will introduce additional complexity, which may undermine the practicality and feasibility of accounting frameworks. Striking an appropriate balance between comprehensiveness and practical applicability will be essential for developing nutrient trading schemes that are both scientifically rigorous and operationally viable.

Additionally, the point and diffuse samples used in these bioassays were collected in Australia and may not be representative globally, depending on the characteristics of watersheds and treatment systems. However, the lack of comprehensive datasets limits the ability to develop universally applicable environmental equivalency ratios. This highlights the need for further research to quantify equivalency for other nutrient sources across diverse geographic contexts. The proposed accounting method in our case study was a common scenario in the Australian context as it involved trading between a downstream point source and an upstream diffuse source. In practice, a point-source credit buyer may meet its nutrient reduction requirement through multiple approaches, including onsite treatment upgrades, operational changes, and partial or full offsetting through externally generated credits when onsite treatments are insufficient or unfeasible. Likewise, a credit seller may generate credits for a specific buyer, sell credits to multiple buyers, or bank credits for future use, depending on the rules of the trading scheme. These arrangements require clear governance for credit certification, temporal validity, monitoring, maintenance, and prevention of credit double-counting. The present study does not prescribe a specific market design for those aspects but provides a biophysical accounting method that could support credit quantification within such trading arrangements. Future research could explore market designs and accounting methods capable of addressing these complexities and ensuring accurate representation of cumulative impacts on receiving waters. However, these multi-party trading frameworks should also balance scientific rigor and practical feasibility.

The delivery ratio (*DR*), co-benefit factor (*BF*), and uncertainty factor (*UF*) have been incorporated into the accounting method primarily as placeholders, rather than being underpinned by rigorous scientific justification for their quantification. This approach reflects the current absence of empirical evidence to support the assignment of values, as these parameters are inherently context-dependent and likely to vary across different trading schemes. Consequently, any attempt to apply uniform values would risk oversimplifying complex ecological and economic interactions. Despite these limitations, disaggregating trading ratios into distinct components, such as equivalency ratio, delivery ratio, co-benefit factor, and uncertainty factor, offers a conceptual advantage. This approach enables a more transparent representation of the underlying assumptions and facilitates refinement as new data becomes available. Furthermore, it provides a structured mechanism for managing uncertainty, reducing the likelihood of arbitrary ratio assignments that lack scientific rigor. By explicitly acknowledging the sources of variability and uncertainty, this accounting method promotes adaptive management and supports the development of more robust, evidence-based trading systems over time.

The successful implementation of nutrient trading between point and diffuse sources depends on a combination of well-designed trading rules, strong economic incentives, and supportive policy and institutional frameworks to ensure that environmental outcomes are achieved. These challenges have been widely acknowledged in previous research, but are beyond the scope of this study. In the present work, nutrient trading between point and diffuse sources is conceptualized as a mechanism to stimulate investment in watershed restoration, an area that has historically been underprioritized due to difficulties in quantifying diffuse nutrient loads and their impacts, as well as challenges with limited financial resources. By incorporating watershed mitigation measures into trading schemes, nutrient trading can serve as a nature-based solution for integrated watershed nutrient management, delivering benefits to both terrestrial and aquatic ecosystems beyond nutrient load management. For watersheds subject to environmental degradation and environmental managers seeking to incentivize restoration efforts, nutrient trading between point and diffuse sources warrants greater attention as a strategy to achieve holistic restoration outcomes. However, realizing these benefits requires more than market-driven economic incentives. It also demands clear policy objectives, regulatory support, and governance structures that prioritize ecological integrity while balancing economic considerations.

## Conclusions

The purpose of nutrient markets is to provide cost-effective pathways to improve environmental health. There is no doubt that the establishment of trading rules and institutional frameworks is critical to the effectiveness of nutrient trading. However, equally important is the establishment of robust nutrient accounting methodologies capable of credibly differentiating the impacts of point and diffuse sources on receiving waters. This study explored a potential nitrogen accounting methodology to improve the scientific foundation for nutrient markets, especially on environmental equivalency ratio determinations. Our exploratory approach integrates both concentrations and loads, equating the environmental impact of different forms of nitrogen delivered in different locations, valuing co-benefits, and accounting for uncertainties. Robust scientific principles need to be applied consistently to build trust and market confidence, with operability to encourage investment in watersheds to achieve environmental benefits. Ultimately, the effectiveness of nitrogen markets in protecting and restoring aquatic ecosystems will depend on a rigorous assessment of outcomes.

## Data Availability

All data used in this study are derived from previously published and publicly available sources upon request. Data sets used for this research are from two published papers as follows: Lu J, Garzon-Garcia A, Chuang A, Burton J, Jackson C, Rogers J, Newham M, Saeck E, Allan M, Burford MA, 2024a. Nutrient metrics to compare algal photosynthetic responses to point and non-point sources of nitrogen pollution. Ecol Indic 158, 111425. https://doi.org/10.1016/j.ecolind.2023.111425. Lu J, Newham M, Chuang A, Burton J, Garzon-Garcia A, Burford MA, 2024b. Factors driving impacts of different nitrogen sources on freshwater and marine green algae. Mar Pollut Bull 208. https://doi.org/10.1016/j.marpolbul.2024.116991. Both papers used a standardized three-day algal bioassay to measure the photosynthetic response of a nitrogen-starved freshwater algal species to nutrients derived from a range of different Australian nitrogen point sources (sewage treatment plants and aquaculture ponds) and eroded soil runoff over a range of concentrations (soil slurries). All datasets have been summarized in Lu et al. (2026). Lu J, Bennett L, Newham M, et al (2026) Nitrogen in urban stormwater caused a significant algal growth response as simulated soil erosion runoff despite differences in nitrogen forms. J Environ Manage 413. https://doi.org/10.1016/j.jenvman.2026.130398. These datasets have been appropriately cited within the manuscript to ensure transparency and reproducibility. No new data were generated or collected specifically for this research.
